# Evaluating atypical language in autism using automated language measures

**DOI:** 10.1038/s41598-021-90304-5

**Published:** 2021-05-26

**Authors:** Alexandra C. Salem, Heather MacFarlane, Joel R. Adams, Grace O. Lawley, Jill K. Dolata, Steven Bedrick, Eric Fombonne

**Affiliations:** 1 Department of Psychiatry, Oregon Health and Science University, Portland, 97239 USA; 2 Computer Science and Electrical Engineering, Oregon Health and Science University, Portland, 97239 USA; 3 Department of Pediatrics, Oregon Health and Science University, Portland, 97239 USA; 4 Department of Medical Informatics and Clinical Epidemiology, Oregon Health and Science University, Portland, 97239 USA

**Keywords:** Autism spectrum disorders, ADHD

## Abstract

Measurement of language atypicalities in Autism Spectrum Disorder (ASD) is cumbersome and costly. Better language outcome measures are needed. Using language transcripts, we generated Automated Language Measures (ALMs) and tested their validity. 169 participants (96 ASD, 28 TD, 45 ADHD) ages 7 to 17 were evaluated with the Autism Diagnostic Observation Schedule. Transcripts of one task were analyzed to generate seven ALMs: mean length of utterance in morphemes, number of different word roots (NDWR), um proportion, content maze proportion, unintelligible proportion, c-units per minute, and repetition proportion. With the exception of repetition proportion (p $= .07$), nonparametric ANOVAs showed significant group differences (p$< 0.01$). The TD and ADHD groups did not differ from each other in post-hoc analyses. With the exception of NDWR, the ASD group showed significantly (p$< 0.01$) lower scores than both comparison groups. The ALMs were correlated with standardized clinical and language evaluations of ASD. In age- and IQ-adjusted logistic regression analyses, four ALMs significantly predicted ASD status with satisfactory accuracy (67.9–75.5%). When ALMs were combined together, accuracy improved to 82.4%. These ALMs offer a promising approach for generating novel outcome measures.

## Introduction

Autism Spectrum Disorder (ASD) is a neurodevelopmental disorder characterized by impairments in communication and social interaction, and restricted and repetitive patterns of behavior^1^. Language differences are prevalent among children with ASD, with most showing some expressive and receptive or pragmatic language impairments^2–5^. Pragmatic, semantic, and syntactic language usage predicts social skills in children with ASD^6^ and pragmatic language impairments have been linked to an increased prevalence of anxiety disorders among children with ASD^7^. Conversational language ability is a significant predictor of job independence and friendship in adults with ASD, both of which are metrics for measuring quality of life^8^. The presence of communication difficulties is a hallmark of autism, and as such are important targets for evaluation and intervention.

Several standardized measures are commonly used for clinical assessment of language in autism, such as the Clinical Evaluation of Language Fundamentals (CELF) and the Peabody Picture Vocabulary Test (PPVT)^9,10^. While such clinical measures offer direct assessment of language ability, they require specially trained professionals for administration, are time- and money-intensive, result in inaccurate performance due to child stress or anxiety, and their limited scope provides only partial examination of linguistic ability^11^. In addition, standardized language testing generates speech samples in highly constrained contexts and lacks ecological validity. One alternative is the parent-reported Children’s Communication Checklist-2 (CCC–2), which is used to assess communication behaviors^12^ and has been shown to accurately identify pragmatic language difficulties in children with ASD^13^. However, more objective language measures are called for, as the fallibility of parent-reported measures is well documented^14,15^.

In contrast to conventional language assessments, measures of expressive language can be generated from natural language sampling, which more accurately reflects an individual’s true verbal communication ability in a real-world context. Barokova and Tager-Flusberg (2020) and others have called for the development of measures which address linguistic heterogeneity among individuals with ASD, specifically citing the utility of natural language samples for their ability to capture differences across a wide range of age and language levels. This methodology uses a spoken language sample to evaluate features like talkativeness, vocabulary, syntax, utterance planning, and articulation^16–18^. Many studies have investigated the validity of such measures to discriminate by age^17,19^ and clinical group^20–23^, and have shown convergent validity with standardized language tests^16^. Expressive language is already well established as a clinical marker of language ability^24–26^.

Many measures of expressive language have been evaluated for their ability to distinguish between ASD and a typically developing (TD) control group. However, because studies have generally not included a non-ASD clinical control condition we cannot know if results of ASD and TD comparisons are specific to ASD. For example, Attention Deficit Hyperactivity Disorder (ADHD) is a neurodevelopmental disorder that has a high co-occurrence with autism, with both groups showing difficulties with language and social communication^27,28^. Children with ADHD show communication and pragmatic differences similar to children with ASD, although with lesser magnitude^29,30^. As ADHD is part of the differential diagnosis of ASD and shows overlap in language domains, it represents a useful comparison group to test how language features in ASD differ from other neurodevelopmental disorders, increasing the ability to examine the specificity of language measures.

Natural Language Processing (NLP) is a broad field at the intersection of computer science, linguistics, and artificial intelligence that aims to analyze speech and language automatically through the use of computational methods. NLP has been used widely in studies examining language use in neurodevelopmental disorders, including autism. Applications range from distributional semantic models used to identify unexpected words in narrative retellings by children with autism^31^ to word alignment models that identify pragmatically inappropriate language in children with ASD compared with their language-matched peers^32^. Other applications include vector-space techniques to detect semantic differences in language of children with ASD and typically developing controls^33,34^. Natural Language Processing is useful for identifying and accurately quantifying characteristics of language in ASD that would be labor-intensive or infeasible to do by hand. As such, there is great potential for using computational methods to produce novel language measures in ASD research; many measures of expressive language can be calculated automatically with appropriate software.

This study was designed to address a gap in the research: namely the power of automatically calculated measures of expressive language to discriminate between ASD and two non-ASD control groups, and the correlation of these measures with common standardized tests. This is part of a larger research project examining language in already-diagnosed children with ASD. Our long-term objectives are to develop quantitative automated tools for outcome measures of language features associated with ASD, though we do not intend the measures presented here to be immediately applicable to screening or diagnosis. This study uses transcripts of a common instrument of autism diagnosis, the Autism Diagnostic Observation Schedule (ADOS) as an expressive language sample. We examined seven measures of expressive language, which were automatically computed for one ADOS activity using NLP methods. Hereafter these are called Automated Language Measures (ALMs) for their ability to be automatically calculated. Our specific goals were to: 1. examine language differences (measured by ALMs) between ASD and two non-ASD control groups (ADHD and TD); 2. analyze the convergent validity of these measures with standardized language measures; 3. investigate the discriminant validity of individual ALMs in classifying ASD status; and 4. examine if gains in discriminant validity could be obtained by combining all ALMs together.

## Methods

### Participants

Participants aged 7 to 17 years with either ASD, ADHD, or TD were recruited for an fMRI study by community outreach and referrals from Oregon Health & Science University’s specialty clinics. Data collection occurred from 2012 to 2018. Potential participants came in for a screening visit to determine if they qualified for the study. During this initial visit, informed written consent or assent was obtained from all participants and their parents. Parents also completed a Developmental and Medical History survey. All children in the ASD and ADHD groups were directly assessed by experienced child psychiatrists and clinical psychologists who confirmed their diagnosis based on DSM-IV-TR criteria^35^. The research diagnostic team reviewed results of the standardized diagnostic assessments (both videos and scored protocols) and used best estimate procedures. ASD was ruled out in the TD and ADHD groups based on the ADOS, clinical interview, and parent-completed autism questionnaires (see below). Exclusion criteria for all groups included the presence of seizure disorder, cerebral palsy, pediatric stroke, history of chemotherapy, sensorimotor handicaps, closed head injury, thyroid disorder, schizophrenia, bipolar disorder, current major depressive episode, fetal alcohol syndrome, Tourette’s disorder, severe vision impairments, Rett’s syndrome, current use of psychoactive medications, non-English speaker, or an IQ below 70.

Of 289 screened participants, 104 were ultimately excluded from this study for not meeting strict diagnostic criteria and another 10 for failing to complete the initial assessment procedures, leaving a sample of 175 subjects (102 ASD, 45 ADHD, 28 TD) included in the main neuroimaging study. Of these, we excluded a further six subjects with ASD due to an untranscribable ADOS session, either for poor sound quality or non-compliance, leaving a total sample of 169 children for this analysis. Sample characteristics for the participants are given in Table 1.

### Instruments

#### Autism diagnosis and severity

The ADOS^36^ is a semi-structured, standardized assessment in which a trained examiner engages participants in activities that are designed to elicit social and communication behaviors indicative of symptoms of ASD as defined in the DSM-IV-TR^35^. In this study, all participants were administered Module 3 of the ADOS-2, designed for children and adolescents with fluent speech. Module 3 comprises 14 tasks that are generally administered in sequence although the tester has some flexibility to change the task order if clinically indicated. All ADOS interviews were administered by research assistants or a senior clinical psychologist trained to research reliability level. All administrations were videotaped and later transcribed. The Social Affect (SA) score (10 items; range 0-20) and the Restricted and Repetitive Behavior (RRB) score (4 items; range 0-8) were used in these analyses^37^. Higher scores indicate more severe ASD symptoms.

Other tests administered were the Autism Diagnostic Interview–Revised (ADI-R), a semi-structured, standardized interview designed to examine three major developmental domains (language and communication, reciprocal social interaction, and restricted, repetitive, and stereotyped behaviors and interests)^38^, and the Social Responsiveness Scale (SRS), a parent-completed measure of autistic symptomatology and associated social impairment suitable for 4–18 year olds^39^. Only caregivers of the ASD group were interviewed with the ADI-R; interviews were administered by trained interviewers. Data were reviewed by the diagnostic team and integrated in the best estimate clinical procedures used to confirm diagnoses.

#### Intellectual level

Intellectual level of participants was estimated with a short form of the Wechsler Intelligence Scale for Children 4th Edition (WISC)^40^. Three subtests were administered: Information, Block Design, and Vocabulary, allowing a full scale IQ to be estimated from the sum of scaled scores of the three subtests according to the formula set out by Sattler and Dumont (2004)^41^.

#### Language assessment

Language characteristics and linguistic pragmatic abilities were assessed using the parent-completed Children’s Communication Checklist, second edition (CCC-2)^12^. The CCC-2 is a widely-used, 70-item standardized checklist of pragmatic and social communication behaviors applicable to children ages 4:0 to 16:11. Caregivers are asked to make a frequency judgment about how often behaviors occur on 4-point scale. The CCC-2 is divided into 10 subscales measuring: (A) speech, (B) syntax, (C) semantics, (D) coherence, (E) inappropriate initiation, (F) stereotyped language, (G) the use of context, (H) non-verbal communication, (I) social relationships, and (J) interests. The first four subscales (A–D) evaluate articulation and phonology, language structure, vocabulary, and discourse; four other subscales (E-H) evaluate pragmatic aspects of communication as well as stereotyped language with atypical or unusual expressions and use of nonverbal communication like facial expressions, bodily movements, and gestures. The last two subscales (I and J) measure behaviors characteristic of children with ASD. Each subscale raw score is converted to age-standardized scores (mean = 10; SD = 3). A General Communication Composite (GCC) is derived by summing scores A to H (mean = 100; SD = 15). A Structural Language scale score is derived by averaging scores A to D, and a Pragmatic Language scale score is obtained by averaging scores E to H. Lower scores are indicative of more problems.

**Table 1:** Sample characteristics.

	ASD	TD	ADHD	p	post-hoc
	$\hbox {n} = 96$	$\hbox {n} = 28$	$\hbox {n} = 45$		
Male sex, N (%)	80 (83.3)	12 (42.9)	31 (68.9)	$< .001$	
Age in years, X (SD)	11.36 (2.21)	11.61 (1.73)	11.46 (1.61)	.84	
Hispanic, N (%)	14 (14.6)	5 (17.9)	3 (6.7)	.53	
Race white, N (%)	77 (82.8)	24 (85.7)	37 (84.1)	.93	
WISC full scale IQ, X (SD)	99.0 (19.7)	113.4 (12.3)	111.6 (13.8)	$<.001$	$ASD < TD, ADHD$
*ADOS scores*					
SA score, X (SD)	9.48 (3.52)	1.04 (1.86)	1.29 (1.44)	$<.001$	$ASD > TD, ADHD$
RRB score, X (SD)	3.47 (1.56)	.52 (.71)	.42 (.58)	$<.001$	$ASD > TD, ADHD$
Total score, X (SD)	12.95 (3.43)	1.56 (2.29)	1.71 (1.67)	$<.001$	$ASD > TD, ADHD$
SRS total t-score, X (SD)	77.27 (10.60)	43.96 (4.14)	53.89 (8.62)	$<.001$	$ASD> ADHD > TD$
*CCC2 scores*					
GCC, X (SD)	73.32 (11.79)	111.96 (8.31)	96.91 (12.78)	$<.001$	$ASD< ADHD < TD$
Structural score, X (SD)	6.50 (2.41)	11.13 (1.12)	9.63 (2.15)	$<.001$	$ASD< ADHD < TD$
Pragmatic score, X (SD)	4.89 (1.82)	11.85 (1.27)	9.17 (1.95)	$<.001$	$ASD< ADHD < TD$

Post-hoc Tukey, $\hbox {p} <.05$. SD: standard deviation. Full ranges of clinical measures can be found in Supplementary Table S1.

### Data

#### Transcription

All ADOS administrations were audio and video recorded. The audio was transcribed according to modified SALT guidelines (Systematic Analysis of Language Transcripts)^42^ by a team of trained research assistants who were blind to the participants’ diagnostic status and intellectual abilities. The ADOS activities Make-Believe and Joint Interactive Play, Description of a Picture, Telling a Story From a Book, Cartoons, Conversation and Reporting, Emotions Conversation, Social Difficulties and Annoyance Conversation, Friends Relationships and Marriage Conversation, and Loneliness Conversation were transcribed. Speech was split into communication units, or c-units, consisting of a main clause and any subordinate, modifying clauses, or of speech fragments that constitute a whole utterance such as responses to questions. Special attention was paid to notation of disfluencies, or mazes, and any unintelligible speech was marked. Any disagreements between transcribers were resolved through discussion with a clinician and a consensus judgment. Transcribers participated in biannual consistency checks to review protocol and ensure continued standardization. Lab transcription guidelines are available upon request from the first author.

The ADOS is a recommended source of natural language for measuring expressive language ability and has been used in previous studies^26,43,44^. We analyzed the transcript of one ADOS task: Friends, Relationships, and Marriage. The focus of this conversation is the participant’s understanding of the nature of personal relationships, on why someone would want to engage in such relationships, and what the participant’s role might be in those relationships. This conversational activity is administered in the second half of the ADOS, after participants have “warmed up” to the testing situation. In addition, this task consistently yielded the most utterances of all ADOS tasks, providing sufficient speech data for analysis. The mean number of participant utterances for the activity was 77.37 (73.1 ASD, 71.4 TD, 90.3 ADHD). The audio had a mean length of 6.27 minutes (6.47 ASD, 5.43 TD, 6.36 ADHD), a satisfactory length for language sampling analysis^45^. This activity also has high standardization of conversational questions, leading to good comparability between participants. The examiner uses pre-established interview questions that are open-ended and designed to facilitate the flow of conversation. Follow-up probes are used at the examiner’s discretion to maintain that flow.

#### Automated language measures (ALMs)

We chose to examine seven expressive language measures that have been explored in previous studies on neurodevelopmental disorders. Mean Length of Utterance in Morphemes (MLUM) and Number of Distinct Word Roots (NDWR) were calculated on all complete, fluent, and intelligible c-units following Gorman et al. (2015). Um proportion, a measure of uh and um usage, was calculated as the total number of *um*s over the total number of *um* + *uh*. Content maze proportion, a measure of disfluency, was calculated as the number of content mazes over the number of content mazes + the number of fillers. Following MacFarlane et al. (2017) we use the term content maze to refer to disfluencies which contain content words (as opposed to the fillers *ah, uh, um, mm, hmm, like, well, you know, I mean*). An example is below:

- **Content maze** (My mom) My dad picked me up.
- **Filler maze** I love (uh) pancakes.

All mazes and fillers were marked during the transcription process, not as a post-hoc coding. Unintelligible proportion was calculated as the number of partially or fully unintelligible c-units over the total number of c-units. C-units per minute (CPM) was calculated as the number of attempted c-units per minute. Repetition proportion is a measure of a child’s repetition of examiner speech, and is calculated as the number of child words that are repeated in a set of two or more from the examiner’s immediately previous turn, divided by the total number of child words. An example is below:

- **Examiner** And the moon was coming up.
- **Child** *The moon was coming up* then.

The child repeats four words from the examiner (“the moon was coming up”) out of five total words, so the repetition proportion is 4/5. A set of repeated words is counted as a repetition if two or more words are exactly identical to the examiner’s speech, or if there is an *approximate match* between the words. Following van Santen et al. (2013), in the case of an approximate match the following were allowed: *you, I, me, we, us* could be substituted with each other; *him/her* and *he/she* could be substituted with each other; *the, a, an, is, are, am, ’m, ’s, is,* and *’re* could be deleted.

These seven ALMs and their calculation methods are summarized in Table 2.

**Table 2:** ALM calculation.

Language construct	Literature source for construct	ALM	Calculation method
Utterance length	Gorman et al. (2015)	MLUM	Mean length of utterance in morphemes in all complete, fluent, and intelligible c-units
Total words	Gorman et al. (2015)	NDWR	Total number of different word roots in all complete, fluent, and intelligible c-units
Uh versus um	Gorman et al. (2016)	Um Proportion	$\frac{{\#}\ \hbox {um}}{{\#}\ \hbox {um} + {\#}\ \hbox {uh}}$
Filler versus content mazes	MacFarlane et al. (2017)	Content Maze Proportion	$\frac{{\#}\ \text {content mazes}}{{\#}\ \text {content mazes} + {\#} \text {fillers}}$
Intelligibility	Abbeduto et al. (2020)	Unintelligible Proportion	$\frac{{\#}\ \text {c-units partially or fully unintelligible}}{\text {total}\ {\#}\ \text {c-units}}$
C-units Per Minute	Abbeduto et al. (2020)	CPM	$\frac{{\#}\ \text {attempted c-units}}{\text {length of task (minutes)}}$
Repetition of others	van Santen et al. (2013)	Repetition proportion	$\frac{{\#}\ \text {words}\ \ge 2\ \text {repeated from examiner}}{\text {total}\ {\#}\ \text {child words}}$

MLUM and NDWR were calculated using software written by Gorman et al. (2015). Um proportion, unintelligible proportion, and c-units per minute were all calculated using software written by the authors. Content maze proportion was calculated using software adapted from MacFarlane et al. (2017)^23^. Repetition proportion was calculated using software from van Santen et al. (2013)^21^. All software was written in Python 2.7^46^.

### Statistical analyses

Our first aim was to explore differences between the three diagnostic groups for the seven ALMs. Because um proportion, unintelligible proportion, and repetition proportion were not normally distributed, standard ANOVA assumptions were violated; we therefore compared groups using nonparametric Kruskal-Wallis one-way ANOVAs. Effect sizes were calculated with eta-squared ($\eta ^{2}$). Significant results ($\hbox {p} < 0.05$) were followed up with post-hoc contrasts using the Games-Howell test.

For the second aim of examining the convergent validity of these ALMs, we calculated the Spearman’s rank correlations between each ALM and key language-related clinical scores from the ADOS and the CCC-2.

Our third aim was to establish the discriminant validity of the ALMs by evaluating their ability to classify ASD status. As post-hoc contrasts indicated that the TD and ADHD groups were not significantly different from each other, these were combined into a non-ASD control group for further analyses. The ALMs had heterogeneous distributions with some approximating normal distributions (MLUM, NDWR, CPM, content maze proportion) and others with extreme skewness (um proportion, unintelligible proportion, repetition proportion). In order to create a common scale, the seven ALMs were recoded as ordinal variables using the observed distribution on the whole sample. Values falling between the 1st and 50th centiles were scored 1, those between the 50th and the 75th centiles were scored 2, values between the 75th and 90th centile were scored 3, and a score of 4 was ascribed to values above the 90th centile. A slight adjustment to this recoding had to be made for um proportion, for which more than 10% of participants had a value of 0. Accordingly, the score of 4 was shifted to the 86th centile to include all zeros; the other score centiles were left unchanged. Scores were reversed for MLUM, NDWR, um proportion, and CPM to account for the inverse nature of those ALMs: lower values on these measures is associated with higher impairment. After recoding, higher impairment is shown by increasing values (from 1 to 4) for all ALMs. Seven binary logistic regression models using ASD status ($0 = \hbox {non}$-ASD; $1 = \hbox {ASD}$) as a dependent variable and each recoded ALM as independent variables were calculated. Models were estimated with and without adjustment on full scale IQ and chronological age. Adjusted models showed consistent superiority in terms of overall significance and proportion correctly classified; accordingly, all models were subsequently adjusted on IQ and age. Their predictive value was evaluated with the Wald statistic. For each model, we also report the -2 log-likelihood, the Nagelkerke pseudo-R square, and the accuracy, alongside sensitivity and specificity. We first estimated a baseline model using only IQ and age as independent variables; models containing each single ALM were subsequently compared to this baseline model to gauge the increment in predictive performance attributable to each ALM.

To address our fourth aim, whether combining the ALMs improves discriminant validity, we estimated a final logistic regression model using all seven ALMs in addition to IQ and age as independent variables. We report the same statistics for this model as for the individual logistic regression models. Finally, we calculated Receiver Operator Characteristic (ROC) curves of the baseline model, the seven individual ALM models, and the combined model with all seven ALMs. For each model, we used the prediction probabilities produced from the logistic regression predictive model. We plotted these nine ROC curves in a graph and report the corresponding area under the curve (AUC).

Following Perneger (1998) and Rothman (2014), we did not use Bonferroni’s adjustment for multiple tests. Throughout, a p-value of $< 0.05$ was retained as a level of statistical significance. All analyses were performed using R statistical computing software^47^.

### Ethical approval

This study was approved by the Oregon Health & Science University Institutional Review Board, Number 00000531, and all research was performed in accordance with their relevant guidelines and regulations.

## Results

### Aim 1: examine language differences between groups

**Figure 1:**
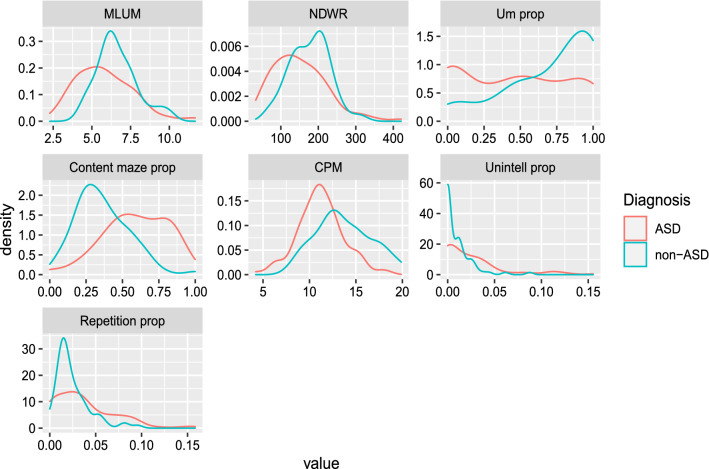
Relative frequency distribution of each ALM by ASD status.

The means and standard deviations of the ALMs for each diagnostic group are shown in Table 3. The relative frequency distribution for each ALM is shown by ASD status in Fig. 1. Nonparametric Kruskal-Wallis one-way ANOVAs showed significant group differences at $p < .01$ for all ALMs except repetition proportion ($p = 0.078$). The largest effect sizes were found for content maze proportion and c-units per minute. MLUM, NDWR, um proportion, and unintelligible proportion each had moderate effect sizes. Repetition proportion had a small effect size. In post-hoc tests, we found significant group differences between the TD and ASD group for all ALMs except NDWR ($p = 0.528$). Significant group differences were found between the ADHD and ASD group for all ALMs. No significant differences were found between the TD and ADHD groups for any of these seven ALMs. Thus, for subsequent analyses we combined the TD and ADHD groups into a non-ASD control group.

**Table 3:** Diagnostic group differences for ALMs.

	ASD		TD		ADHD		p-value	$\eta ^2$	post-hoc
	Mean	SD	Mean	SD	Mean	SD			
MLUM	5.808	1.858	6.772	1.462	6.522	1.242	0.002253	0.0614	$TD, ADHD > ASD$
NDWR	150.802	73.175	162.500	41.320	186.222	56.754	0.001798	0.0641	$ADHD > ASD$
Um prop	0.455	0.367	0.714	0.351	0.691	0.277	0.000154	0.0937	$TD, ADHD > ASD$
Content maze prop	0.593	0.224	0.352	0.141	0.369	0.204	6.424e-10	0.2430	$TD, ADHD < ASD$
Unintell prop	0.026	0.034	0.008	0.014	0.010	0.016	0.001021	0.0709	$TD, ADHD < ASD$
CPM	11.211	2.492	12.978	2.321	14.107	3.268	1.295e-06	0.1513	$TD, ADHD > ASD$
Repetition prop	0.037	0.034	0.025	0.017	0.024	0.020	0.07831	0.0186	$TD, ADHD < ASD$

P-value determined by Kruskal-Wallis test. Eta-squared ($\eta ^2$) effect sizes were calculated for each Kruskal-Wallis result. Post-hoc analysis performed by Games-Howell test.

The direction of differences between diagnostic groups fall into two sets. The ASD group had significantly higher means of content maze proportion, unintelligible proportion, and repetition proportion (where higher values are more characteristic of ASD) and significantly lower means of MLUM, NDWR, um proportion, and CPM (where lower values are more characteristic of ASD). This pattern is consistent with the expectation for increased language atypicalities in the ASD group.

We retained the ALM repetition proportion in later analyses despite it falling short of statistical significance in the Kruskal-Wallis test for two reasons: 1. repetitive speech has a high theoretical significance in language characterization in ASD and 2. it was possible that it would become a significant predictor once IQ, age, and other ALM scores were covaried.

### Aim 2: convergent validity of ALMs

In order to evaluate the convergent validity of these ALMs, we evaluated how well they correlated with standardized clinical measures. We calculated Spearman rank correlation coefficients between each of the seven ALMs and the child’s CCC-2 and ADOS scores. From the CCC-2 we used the General Communication Composite (GCC), Structural score, and Pragmatic score. From the ADOS, we used the total Social Affect (SA) score and total Restricted and Repetitive Behavior (RRB) score. These results are summarized in Table 4.

**Table 4:** Relationship of ALMs to clinical scores using Spearman correlations.

	MLUM	NDWR	Um	Content maze	Unintell	CPM	Repetition	CCC2	CCC2	CCC2	ADOS	ADOS
			Prop	Prop	Prop		Prop	GCC	Structural	Pragmatic	RRB	SA
MLUM	–											
NDWR	**0.74**	–										
Um prop	0.14	0.04	–									
Content maze prop	− 0.08	− 0.05	**− 0.39**	–								
Unintell prop	*− 0.17*	**− 0.20**	− 0.06	**0.28**	–							
CPM	**0.22**	**0.50**	− 0.01	− 0.08	− 0.06	–						
Repetition prop	**− 0.19**	**− 0.29**	**− 0.22**	*0.16*	**0.19**	**− 0.25**	–					
CCC2 GCC	**0.31**	**0.26**	**0.29**	**− 0.47**	**− 0.25**	**0.29**	− 0.10	–				
CCC2 Structural	**0.33**	**0.28**	**0.27**	**− 0.46**	**− 0.25**	**0.26**	− 0.11	**0.95**	–			
CCC2 Pragmatic	**0.26**	**0.23**	**0.29**	**− 0.44**	**− 0.23**	**0.32**	− 0.09	**0.95**	**0.82**	–		
ADOS RRB	**− 0.22**	− 0.11	**− 0.26**	**0.46**	**0.23**	**− 0.25**	0.05	**− 0.64**	**− 0.54**	**− 0.68**	–	
ADOS SA	**− 0.43**	**− 0.43**	**− 0.29**	**0.46**	**0.26**	**− 0.45**	**0.20**	**− 0.72**	**− 0.64**	**− 0.73**	**0.63**	–

Italics indicates significance with $.01<p <.05$. Boldface indicates significance with $p<.01$.

With the exception of repetition proportion, all ALMs were significantly correlated with all three scores from the CCC-2, with absolute values of correlation coefficients ranging from .23 to .47. The direction of correlations followed the same groupings seen previously: content maze proportion, unintelligible proportion, and repetition proportion were negatively correlated with the CCC-2 scores. MLUM, NDWR, um proportion, and c-units per minute were all positively correlated with the CCC-2 scores. This was the expected direction of correlation, since lower scores on the CCC-2 indicate language difficulties which are associated with ASD. Notably, content maze proportion had the strongest association with the three CCC-2 scores (absolute value range .44 to .47). Conversely, repetition proportion had small and insignificant correlations with CCC-2 scores.

All ALMs were significantly correlated with the ADOS total SA score, with absolute values of correlation coefficients ranging from .20 to .46. Five ALMs were significantly correlated with the ADOS total RRB score, with absolute values of correlation coefficients ranging from .22 to .46, while NDWR and repetition proportion were not significantly correlated with the RRB score. Overall, RRB score correlations were of smaller magnitude than those with the SA score. The direction of correlations followed the same groupings seen previously. This was expected since higher ADOS SA and RRB scores are associated with an ASD diagnosis.

### Aim 3: discriminant validity of ALMs

We then examined the discriminant validity of these seven ALMs to determine their ability to distinguish diagnostic groups by using logistic regression models. We excluded 10 participants from this analysis (7 ASD, 3 non-ASD): six participants with undefined um proportion (for whom *um* and *uh* did not occur in the Friends and Marriage Conversation), and four participants who did not have WISC IQ results. These 10 participants were excluded from all models to ensure comparability, leaving a sample of 89 ASD and 70 non-ASD. Results of the logistic regression models are summarized in Table 5.

Model 0 is the baseline model, using IQ and age as independent variables. Models 1-7 are logistic regression models using a single recoded ordinal ALM as well as IQ and age as independent variables.

**Table 5:** Logistic regression models for ALMs adjusted by age and IQ.

Model		Goodness of fit		Wald test		Classification			
Name	Variable	-2 Log Like	Nagelkerke $R^2$	Wald	p-value	Accuracy	Specificity	Sensitivity	AUC
Model 0	IQ and Age	196.5606	0.1701			0.6289	0.7191	0.5143	0.6868
(Baseline)	IQ			16.683	4.417e-0				
	Age			0.006	0.9382				
Model 1	MLUM	185.2495	0.2504	0.4865	0.9219	0.6415	0.6404	0.6429	0.7120
Model 2	NDWR	193.7276	0.1907	2.5024	0.4749	0.6352	0.6517	0.6143	0.6937
Model 3	Um prop	179.1942	0.2911	14.8426	0.0020	0.6792	0.7191	0.6286	0.7600
Model 4	Content maze prop	163.5166	0.3896	15.5078	0.0014	0.7547	0.7640	0.7429	0.8149
Model 5	Unintell prop	181.0161	0.2790	12.2279	0.0066	0.6792	0.6742	0.6857	0.7518
Model 6	CPM	176.5453	0.3084	15.6164	0.0014	0.6918	0.7191	0.6571	0.7722
Model 7	Repetition prop	188.4896	0.2280	7.6232	0.0545	0.6792	0.7528	0.5857	0.7222
Model 8	All 7	105.2476	0.6767			0.8239	0.8286	0.8202	0.9223
	MLUM			2.0553	0.5610				
	NDWR			4.1463	0.2461				
	Um prop			3.2571	0.3537				
	Content maze prop			10.3446	0.0159				
	Unintell prop			10.1375	0.0174				
	CPM			14.1226	0.0027				
	Repetition prop			6.9012	0.0751				

Model 0 uses only IQ and age. Models 1-7 use a single ordinal recoded ALM, and include IQ and age. Model 8 uses all seven recoded ALMs, and includes IQ and age. -2 Log Like is the -2 times the log-likelihood of the model (low values reflect better fit). Nagelkerke $R^2$ is a pseudo-$R^2$ measure (high values reflect better fit). Wald is the Wald test statistic chi-squared value and p-value is the p-value of the Wald result for the listed variable. The degrees of freedom for the Wald test were 1 for IQ and age and 3 for all other variables. Accuracy, specificity (true negative rate), sensitivity (true positive rate), and AUC are classification results for predicting ASD diagnosis.

The baseline model 0 classification accuracy was 62.89%. As shown by the Wald test statistic, this effect was largely driven by IQ. Age did not significantly contribute to the model; however, we adjusted all subsequent models on both IQ and age as they are developmentally relevant metrics. Models 1-7 had classification accuracy values ranging from 63.52% for NDWR to 75.47% for content maze proportion. The model for content maze proportion had the highest accuracy out of the seven models by over five percentage points.

Specificity (the true negative rate) was 71.91% at the baseline model, reflecting the strong effect of IQ. However, sensitivity (the true positive rate) was only 51.43%. The models for MLUM and NDWR were not significant (Wald test: $p >.45$) and resulted in very modest improvements in sensitivity while having lower specificity compared to the baseline model. By contrast, the models for um proportion, content maze proportion, unintelligible proportion and c-units per minute each had significant Wald test statistics ($p < .01$). Improvements in sensitivity were attained in each of these four models; however, only content maze proportion resulted in gains in specificity as well. The ALM repetition proportion fell just short of significance (Wald test: $p = .0545$). Remarkably, this model had one of the highest levels of specificity (75.28%) of all ALM models though it remained poor for sensitivity (58.57%).

### Aim 4: combined power of ALMs to increase discrimination between groups

Lastly, we estimated a logistic regression model using all seven recoded ordinal ALMs as well as IQ and age as independent variables and ASD status as the dependent variable (see model 8 in Table 5). This model had the highest classification accuracy of all models, 82.39%. It also had the highest specificity and sensitivity of 82.86% and 82.02%, respectively. Only three ALMs reached significance in the Wald test statistic: content maze proportion, unintelligible proportion, and c-units per minute. This indicates that each of these three ALMs uniquely improved the prediction over and beyond the effect of the other six ALMs and the covariates of age and IQ. The ALMs which did not significantly contribute to their respective individual models—MLUM, NDWR, and repetition proportion—also did not significantly contribute to the combined model. Additionally, um proportion lost the significance it had in its own model when combined with the other ALMs.

**Figure 2:**
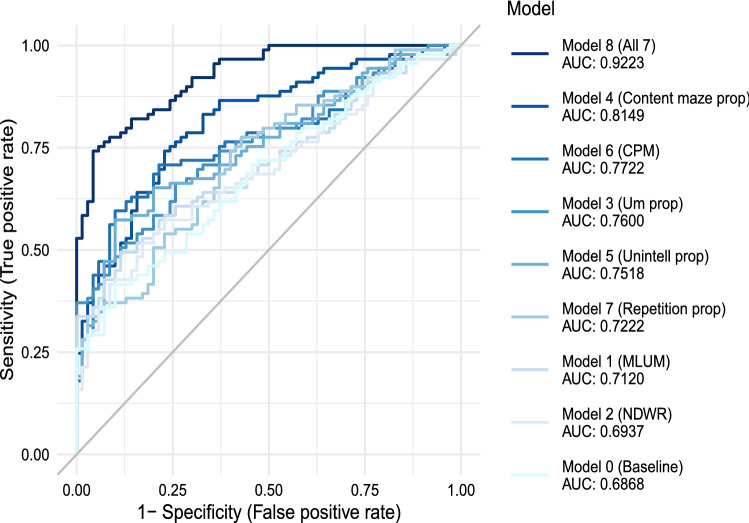
ROC curve for logistic regression models, evaluated on class probabilities. AUC is area under the curve. Baseline is modeled with only IQ and age as independent variables. All other models are adjusted on IQ and age.

We estimated ROC curves for all models in Table 5. In these calculations, we used the class prediction probabilities estimated from the logistic regression models as the predictors in the ROC curves. The resulting nine ROC curves are shown in Fig. 2, alongside their AUC values. The combined model (model 8) had the highest AUC of 0.9223, whereas the baseline model (model 0) had the lowest AUC of 0.6868.

## Discussion

We calculated seven automated language measures from the transcripts of one task of the ADOS. Six measures showed significant differences between ASD and a neurodiverse control group. Consistent with the high prevalence of language atypicalities in autism, the ASD group had significantly lower means of MLUM, um proportion, and c-units per minute than the TD and ADHD groups, and significantly lower means for NDWR than the ADHD group. The ASD group had significantly higher means of content maze proportion, unintelligible proportion, and repetition proportion than the TD and ADHD groups. There were no statistical differences between the TD and ADHD groups for any ALM. Furthermore, ALMs that discriminated between ASD and TD also discriminated between ASD and ADHD, suggesting that these ALMs capture features of language specific to ASD, and not simply those common to all neurodevelopmental disorders. Content maze proportion and c-units per minute were the most discriminant measures as shown by larger effect sizes in Kruskal-Wallis ANOVAs while MLUM, which measures syntactic complexity, and NDWR, which measures vocabulary size, differed only modestly across groups. Like the other ALMs, repetition proportion showed means for the ASD group that were indicative of more atypicality than for the other two groups, but differences fell just short of statistical significance. One plausible explanation for this result is the reduced statistical power due to the low base rate of child-examiner repetitions in our experiment. It is possible that analyses of longer transcripts (such as several or all ADOS tasks) or of more naturalistic speech samples would improve the sensitivity of repetition proportion to ASD anomalies.

The convergent validity of the ALMs was implied by small but significant Spearman rank correlations between the ALM scores and the clinical measures CCC-2 and ADOS, confirming that these ALMs are capturing language differences commonly seen in clinical groups. All ALMs except repetition proportion were significantly correlated with the General Communication Composite (GCC), the Structural score, and the Pragmatic score of the CCC-2. All ALMs were significantly correlated with the ADOS Social Affect (SA) score, and all except NDWR and repetition proportion were correlated with the ADOS Restricted and Repetitive Behavior (RRB) score. The clinical measure most correlated with the ALMs was the ADOS SA score (absolute value range .26 to .46; median .43). The smaller magnitude of RRB score correlations (absolute value range .05 to .46; median .23) is consistent with the fact that the SA score is measuring socio-communicative behaviors whereas the RRB score is more driven by non-language features of autism such as sensory-motor mannerisms or cognitive-behavioral inflexibility. Overall, the magnitudes of Spearman correlations were modest; none of the absolute values were above 0.5, which is a common rule of thumb for moderate correlation. It is notable, however, that clinical scores were provided by highly trained professionals spending 45-60 minutes with a participant or by parents who have observed their child over time and across contexts. In that respect, it is remarkable that ALMs computed on only six minutes of language correlated with those conventional scores.

In our investigation of discriminant validity, four ALMs made significant contributions to their individual logistic regression models as independent variables and achieved higher accuracy and sensitivity than a baseline model. Repetition proportion followed the same pattern, but did not quite reach significance. All ALMs increased the sensitivity, or true positive rate, of their model, resulting in more participants with ASD being correctly classified as having ASD. Content maze proportion and repetition proportion were the only ALMs that resulted in higher specificity than the baseline model 0. Those two ALMs were thus better than IQ and age alone at correctly identifying participants without ASD, since the proportion of true negatives, or participants correctly classified as non-ASD, improved. Overall, content maze proportion achieved the best sensitivity and specificity, with an overall accuracy of over 75%. Thus, um proportion, content maze proportion, unintelligible proportion, and c-units per minute each showed discriminant validity by being able to distinguish children with ASD from typically developing children and children with ADHD. An anonymous reviewer asked if the group differences we report could be attributed to the proportionally larger number of females in the TD group as compared to the ASD and ADHD groups. To address this issue, we estimated new versions of these logistic regression models with child sex as a covariate. We found that while sex was a significant covariate in most of the models, it did not change the significance of the ALMs in any of the models.

Since some ALMs made improvements to specificity while others improved only sensitivity, we investigated the discriminant validity of using all the ALMs together to differentiate children with and without ASD. When the ALMs were combined in one logistic regression model (model 8), overall accuracy improved substantially (82.4%), with high levels of specificity (82.9%) and sensitivity (82.0%) as well. Content maze proportion, unintelligible proportion, and c-units per minute significantly improved prediction of ASD status over and beyond the effect of other ALMs and of covariates. Of the four ALMs that were contributory in their own models, one (um proportion) lost statistical significance when combined with the other ALMs. This very likely reflects the co-linearity between um proportion and content maze proportion in model 8 (Spearman: -.39) due to the fact that both ALMs are conceptually similar measures of disfluency.

NDWR and MLUM remained non-contributory in model 8, and may not be relevant to ASD, however these ALMs could still be useful in a less verbal sample. Repetition proportion did not reach statistical significance ($p = .075$); as explained above, this may simply reflect its low sensitivity when measuring an infrequent language characteristic. Further analyses will determine if other and longer language samples would improve its performance; likewise, this ALM might be more relevant when used with samples of younger or more developmentally delayed participants whose language may be more repetitious. Thus, performance of this ALM remains to be tested in different samples.

The AUC values for the logistic regression prediction probabilities followed a similar pattern as accuracy, as shown in Table 5. Model 8 had the highest AUC of 0.9223, followed by model 4 (content maze proportion) which had an AUC of 0.8149. Model 0 had the lowest AUC of 0.6868. Notably, all AUC values are higher than their corresponding accuracy values. One plausible contributing factor is that the accuracy represents the logistic regression results for a cut-off value of 0.5. However, that is not necessarily the optimal cut-off for the prediction probabilities. The ROC curve instead calculates the true positive rate and false positive rate across many thresholds, and can thus capture results not only at 0.5, but at the optimal cut-off and others in between. These AUC values alongside the other classification results again demonstrate the discriminant validity of several of the ALMs. Based on the findings of our study sample, content maze proportion, unintelligible proportion, and c-units per minute stand out as the best ALMs to use together to distinguish between ASD and a neurodiverse control group.

Content maze proportion, a measure of disfluency, was the highest performing ALM according to many different metrics. It significantly differentiated ASD and non-ASD in a Kruskal-Wallis ANOVA, and had the largest eta-squared effect size of 0.2430. It showed the highest correlations with measures from both the CCC-2 and the ADOS (absolute value range 0.44 to 0.47), a similarly strong correlation as found by Abbeduto et al. (2020) in a fragile X sample between their measure of disfluency (a proportion of the total number of c-units that include a maze or verbal disfluency) and the standardized measures CELF, VABS (Vineland Adaptive Behavior Scales), and GFTA (Goldman-Fristoe Test of Articulation). Of the seven individual logistic regression models, it had the best fit according to both -2 log likelihood and Nagelkerke $R^2$, and the highest accuracy, specificity, and sensitivity. It also contributed significantly to model 8. To the authors’ knowledge, only two prior studies have examined content mazes^23,48^. The lack of published literature on content mazes combined with the high performance of content maze proportion in this study indicate that it should be explored further. This ALM shows particular promise as a measurable language differential between ASD and non-ASD groups.

This study has several limitations. These results are constrained by our sample, which has a large age range (7-17 years), relatively high IQ (average 99-111 across clinical groups), and unequal group sizes. While age and IQ were included as co-variates in the logistic regression models, using a more tightly controlled sample may produce more robust results. While these seven ALMs showed themselves to be useful measures of expressive language, they are still relatively exploratory measures and could be refined further. Alternative calculations of some ALMs such as finding mean length of utterance in words^24,49^ or including incomplete or disfluent utterances, could change the performance of MLU. Likewise, NDWR could be more robust if it were calculated as type-token ratio instead^50^, or if it were replaced with a count of the total number of words. Um proportion could be calculated using different weightings^51^. While high reliability with by-hand calculation has been established for MLUM and content maze proportion, it has not yet been verified for the other ALMs^23,52^. The ALMs unintelligibility proportion and repetition proportion are of limited utility in our study because a large number of participants were at the floor of these measures, making them difficult to accurately analyze compared with the other measures. The ALM um proportion is limited in scope because a small number of participants have an undefined result due to the nature of the calculation. The ordinal recoding for the logistic regression models has limitations: the recoding was done to calibrate all of the ALMs to the same scale, but the choices of cut-offs for recoding were somewhat arbitrary. Another limitation lies in manual transcription, which is time consuming and costly. However, progress in voice recognition technology should help to bypass this step soon.

There are many future directions for this work. The ADOS is used widely, making our methodology readily replicable and accessible to many labs. MacFarlane et al. (2017) found ADOS activity to be a robust predictor of disfluency use, and Abbeduto et al. (2020) showed higher correlations between expressive language measures and clinical assessments in a narration task than in a conversation task. This suggests that using a different ADOS task, such as one focused on narrating a picture, may be a more robust way to measure the utility of our ALMs; we plan to examine this possibility in future work. Additionally, while establishing the convergent and discriminant validity of the ALMs is a first step towards establishing them as language outcome measures, more work is needed. Future steps for this process would include evaluating their test-retest reliability, consistency across different expressive language samples, and responsivity to real change. Finally, ALMs should be tested as objective, quantifiable measures of atypical language in samples of younger or more language impaired participants where they might contribute to novel screening and diagnostic tools that harness new technologies.

## Conclusions

We applied computational methodology to a clinical speech corpus and quantified expressive language through Automated Language Measures. We established moderate convergent validity and good discriminant validity for these ALMs, with content maze proportion and c-units per minute showing especially promising results in discriminating children with ASD. ALM calculations are blind to participant status unlike most clinical measures, such as the CCC-2 and the ADOS, which can be influenced by the parent or professional’s prior knowledge of the child. Our results confirm the potential of using Natural Language Processing for evaluating language samples in ASD research. Compared to conventional measures of language analysis, NLP has the strong advantage of being automated, reliable, fast, and applicable across various age groups and sampling contexts. We do not yet expect these measures to be used as an alternative method of language evaluation. Instead, we intend this work to be a proof of concept of using Automated Language Measures to learn more about language impairment in children with ASD, without depending upon extensive language testing or biased parent reports. We see great value in further development of Automated Language Measures to aid in characterizing language in ASD and evaluating outcomes.

## Supplementary information

Supplementary material 1 (pdf 77 KB)
