# Supplementary material for: Evaluating atypical language in autism using automated language measures
Source: Sci Rep. 2021 May 26;11:10968. doi: 10.1038/s41598-021-90304-5 (PMC8155086; doi:10.1038/s41598-021-90304-5)
Supplement: Supplementary file 1 — Supplementary material 1 (pdf 77 KB) [file 41598_2021_90304_MOESM1_ESM.pdf]

Evaluating atypical language in autism using automated language measures: Supplementary Information

Alexandra C Salem, Heather MacFarlane, Joel R Adams, Grace O Lawley, Jill K Dolata, Steven Bedrick, Eric Fombonne

Supplementary Table S1: Expanded sample characteristics

	**ASD (n=96)**				**TD (n=28)**				**ADHD (n=45)**			
	MIN	MAX	MEAN	SD	MIN	MAX	MEAN	SD	MIN	MAX	MEAN	SD
**Age in years**	7.80	17.34	11.36	2.21	8.75	15.36	11.61	1.73	8.55	14.17	11.46	1.61
**WISC full scale IQ**	40.00	138.00	99.03	19.67	90.00	140.00	113.36	12.28	86.00	138.00	111.60	13.85
**ADOS SA score**	3.00	20.00	9.48	3.52	0.00	8.00	1.04	1.86	0.00	4.00	1.29	1.44
**ADOS RRB score**	0.00	8.00	3.47	1.56	0.00	2.00	0.52	0.71	0.00	2.00	0.42	0.58
**ADOS total score**	7.00	23.00	12.95	3.43	0.00	10.00	1.56	2.29	0.00	6.00	1.71	1.67
**SRS total t-score**	51.00	101.00	77.27	10.60	37.00	51.00	43.96	4.14	41.00	78.00	53.89	8.62
**CCC-2 GCC score**	45.00	103.00	73.32	11.79	92.00	125.00	111.96	8.31	69.00	125.00	96.91	12.78
**CCC-2 Structural score**	1.00	12.00	6.50	2.41	8.75	12.50	11.13	1.12	3.75	12.50	9.63	2.15
**CCC-2 Pragmatic score**	1.50	10.75	4.89	1.82	8.75	13.75	11.85	1.27	5.75	13.75	9.17	1.95

Supplementary Table S2: Expanded ALM diagnostic group differences

	**ASD (n=96)**				**TD (n=28)**				**ADHD (n=45)**			
	MIN	MAX	MEAN	SD	MIN	MAX	MEAN	SD	MIN	MAX	MEAN	SD
**MLUM**	2.279	11.674	5.808	1.858	4.541	9.962	6.772	1.462	4.163	10.098	6.522	1.242
**NDWR**	33	421	150.802	73.175	68	217	162.500	41.320	63	317	186.222	56.754
**Um prop**	0	1	0.455	0.367	0	1	0.714	0.351	0	1	0.691	0.277
**Content maze prop**	0	1	0.593	0.224	0.071	0.769	0.352	0.141	0.059	1	0.369	0.204
**Unintell prop**	0	0.155	0.026	0.034	0	0.062	0.008	0.014	0	0.088	0.010	0.016
**CPM**	4.205	18.345	11.211	2.492	9.367	18.177	12.978	2.321	8.079	19.872	14.107	3.268
**Repetition prop**	0	0.158	0.037	0.034	0	0.083	0.025	0.017	0	0.096	0.024	0.020
